# Evaluation of the Health and Nutritional Status of Discharged Children From Malnutrition Treatment Centres Using Mobile Phone Calls During the COVID-19 Lockdown in Jharkhand, India

**DOI:** 10.7759/cureus.38314

**Published:** 2023-04-29

**Authors:** Kumari Asha Kiran, Manisha Kujur, Reema Kumari, Vidya Sagar, Dewesh Kumar, Gashaw Hassen, Vivek Kashyap, Ajit K Prasad, Rishabh K Rana

**Affiliations:** 1 Preventive Medicine, Rajendra Institute of Medical Sciences, Ranchi, Ranchi, IND; 2 Preventive Medicine, The State Center of Excellence for Management of Severe Acute Malnutrition, Ranchi, IND; 3 Community Medicine/Preventive and Social Medicine, Rajendra Institute of Medical Sciences, Ranchi, Ranchi, IND; 4 Internal Medicine, University of Maryland Capital Region Medical Center, Largo, USA; 5 Medicine, Addis Ababa University, Addis Ababa, ETH; 6 Progressive Care, Mercy Medical Center, Baltimore, USA; 7 Medicine and Surgery, Parma University, Parma, ITA; 8 Community Medicine, Rajendra Institute of Medical Sciences, Ranchi, Ranchi, IND; 9 Family and Community Medicine, National Health Mission, Ranchi, IND; 10 Preventive and Social Medicine/Community Medicine, Shaheed Nirmal Mahto Medical College and Hospital (Erstwhile Patliputra Medical College), Dhanbad, IND

**Keywords:** tribal population, mobile phone follow-up, integrated child development services, malnutrition treatment centre, severe acute malnutrition

## Abstract

Background

The Indian state of Jharkhand has the highest rate of wasting (29%) among young children. Mobile audio call follow-up can be used to assess such children with severe acute malnutrition (SAM).

Aim

This study evaluated SAM children during the COVID-19 outbreak and learn more about the status of their home/community care, and caregivers’ awareness of integrated child development services (ICDS) and COVID-19 prevention.

Methods

Contact numbers of caregivers for discharged children were obtained from 54 malnutrition treatment centers (MTCs). In April and June 2020, mentors conducted follow-up interviews using mobile phone calls.

Results

Seven children (1.72%) were reported dead and 400 were alive, mostly girls (59.5%). Only a few caregivers observed post-discharge ailments (15.4%) and weight loss (7.7%) in their children. Children aged six to 24 months were characterized by continued breastfeeding (88.0%) at most five to six times a day (45.8%). Most of the children were not fed as per maternal infant and young child feeding protocols. Age in months with an adjusted odds ratio (OR) of 0.55 (1.00-1.11) as a 95% confidence interval (CI), age category, with an adjusted OR of 4.32 (1.71- 10.94) as 95% CI, and breastfeeding with adjusted OR 1.85 (1.07- 3.21) as 95% CI were three major predictors for a well-fed child.

Conclusion

Community involvement is crucial in the follow-up of children with SAM for effective rehabilitation. Mobile phone audio call follow-up is a relatively cost-effective approach to tackle geographic barriers and COVID-19 lockdown-induced situations. There are major gaps mainly in informing caregivers on how to manage COVID-19 with breastfeeding.

## Introduction

Undernutrition including wasting, stunting, being underweight, and micronutrient deficiencies-kills 45% of children under five in low- and middle-income countries [1]. India accounts for over 50% of global child malnutrition [2]. Protein-energy malnutrition (PEM) in low-income nations predisposes children to serious infectious illnesses. Severe childhood malnutrition stunts thymic growth, lowering peripheral lymphocyte numbers and lowering immunity. Nutrient-acquired immunodeficiency syndrome results [3].

The lymphocytic derivatives that provide acquired immunity and the host defense mechanism mediated by macrophages and granulocytes are reduced in severely undernourished children, leaving them more susceptible to opportunistic infections [4]. Acute malnutrition can cause complicated socioeconomic issues [5, 6]. Jharkhand actively fights malnutrition. The Jharkhand Government and the National Health Mission (NHM) established nutrition rehabilitation centers (NRCs) known across the state as malnutrition treatment centers (MTCs). The state has set up 96 district MTCs for SAM and other wasting children. Children are followed up four times in 25 days after leaving MTCs. MTCs periodically reassess a child's nutrition and health during follow-up visits [7, 8]. Post-discharge follow-ups have improved malnourished children's growth. MTCs treat SAM kids till they gain 15% of weight while they are admitted to the centers. In NRCs/MTCs apart from treatment of the ailments, specially prepared food like ready-to-use therapeutic food (RUTF) locally termed F-100, and F-75 is given [9, 10].

The 2016 Indian National Family Health Survey (NFHS) and other studies show Jharkhand has the highest percentage of child wasting (29%) in the country. According to the World Health Organization (WHO) growth charts, 11.4% of children aged 0-59 months in Jharkhand are severely wasted owing to malnutrition (WHZ-3 of the median). 

Jharkhand's basic nutrition services, particularly SAM therapy for children, were severely affected by the COVID-19 pandemic. The economic slowdown, health system instability, food insecurity, and household income loss could increase the risk of malnutrition. Current estimates imply a COVID-19-induced dietary crisis might raise global SAM prevalence by 14.3%. The sudden lockdown with movement restrictions forced children in MTCs to leave before treatment was finished or prevent them from returning for follow-up. SAM parents often live in poverty or extreme poverty; therefore, the lockdown meant losing daily revenue for families below the poverty line [1, 6, 10]. The lockdown has halted tuberculosis (TB) elimination, normal immunization, and nutritional treatment [11, 12]. Evidence implies studies are not robust enough to evaluate SAM children over a long time. These children should be evaluated in large groups for longer periods. More research is needed once SAM children are out of MTCs and back in their communities [13, 14, 15].

The Rajendra Institute of Medical Sciences, the state's top medical school, hosts the State Centre of Excellence for Management of Severe Acute Malnutrition (SCoE-SAM) in conjunction with the Department of Health, Medical Education, and Family Welfare, the Government of Jharkhand, and UNICEF [16]. Researchers at SCoE-SAM with the Department of Preventive and Social Medicine (PSM), Rajendra Institute of Medical Sciences (RIMS), Ranchi, collaborated with the Health Department, Government of Jharkhand, and UNICEF to study follow-up and transportation during the lockdown. Since all SAM children enrolled with the State Portal for SAM Children had their parents mobile numbers made available, tracking them through their parents' phones was a no-brainer. Recent evidence suggests there has been a four-fold growth in Indian smartphone users, including rural ones [15, 17, 18].

This study followed SAM children across the state to evaluate the general health status of children discharged from MTC during COVID-19, measure the awareness of mothers and carers, and provide nutrition-related messaging and counseling.

## Materials and methods

RIMS, Ranchi Institutional Ethical Committee approved the study. The interviewers were district-run MTC faculty and mentors and state and district event resource persons. The SAM children were admitted until March 21, 2020, and discharged soon after India's COVID-19 lockdown. Overall 15 mentors who agreed to participate in this study were chosen for telephonic follow-up. After Department of Health monitoring, 33 MTCs were operational on April 15, 2020. The state government's database website provided all such children's names, genders, localities, districts, and parents' mobile phone numbers. Fifty-four MTCs populated the online database of discharged children from March 1, 2020, to June 30, 2020, along with parent and frontline worker contact information. Interviewers called MTC-discharged children's parents. Telephonic or mobile phone interviews are simple, but interviewers must address miscommunications, ethical issues, and language barriers [19, 20, 21]. The State Centre of Excellence based in RIMS trained all mentors on interviewing. Before collecting data, the purpose of the call was explained. After verbal consent from parents or carers, information was gathered using the three principles of active listening with patience and non judgment, understanding themes with empathy, and addressing issues. Interviewers were also told to call from a private location. Overall 15 mentors received 1014 MTC-discharged child data sets. In April and June 2020, each mentor called 100 database phone numbers to gather SAM children's health and nutrition data. Hilly Jharkhand has poor connectivity. Each mentor was advised to be patient when calling their subjects due to expected connectivity and call response issues, such as first-call failures, non-responses, wrong numbers, and switched off phones. Connectivity and call-answering issues resulted in four to five call attempts.

Questionnaire

The Department of Preventive and Social Medicine and UNICEF created a questionnaire to capture the study's components. A piloting was done for checking the internal and external validity of the questionnaires over a mobile phone. After correcting minor phrasings we modified the questionnaire for final use. The Hindi Google Forms tool allowed interviewers to record their responses. Local language interviews were encouraged by mentors. The responder was asked about the SAM child's age, birth order, siblings, etc. Qualitative and subjective responses gave a general picture of the child's health and nutrition status. Questions were based on six thematic areas: 1) basic details; 2) breastfeeding if the child is under two years; 3) current health of child & nutrition status of children & knowledge of parents regarding nutrients; 4) complementary feeding; 5) routine immunization; and 6) COVID-19 appropriate behavior. Based on the mentor's expertise and local customs, subjects were called between 7 a.m. and 8 p.m.

Inclusion criterion

Responders who provided child details and consented to their use in future research were included in the final data collection and processing.

Data analysis

Google Forms were downloaded as MS Excel sheets and analyzed using SPSS (IBM Corp. Released 2010. IBM SPSS Statistics for Windows, Version 19.0. Armonk, NY: IBM Corp). Quantitative and qualitative data were presented as mean, standard deviation, and percentages. Chi-square tests measured the association, and p-values under 0.05 were significant. The Poisson regression predicted ordinal dependent variables.

## Results

Overall 407 consenting participants provided 407 responses. Parents reported seven children had died. Data collected from 400 alive children were analyzed to determine their health, family structure, and key responses. We verified mobile number owners in the database. We inquired about awareness regarding COVID-19. Auxiliary nursing midwives (ANMs) and "Sahiyas"- Jharkhand's Accredited Social Health Activists (ASHA's) also participated. They called the parents repeatedly to obtain accurate responses.

Age categories

Zero to six months had a mean age of 3.71 months 1.61, >6 months-24 months had 15.76 months 4.81, and >24 months had 39.88 months 11.41. The overall mean age was 20.91 months ±12.86. Most SAM children (71%), and those who died (85.7%), were between six and 24 months old.

Sociodemographic characteristics of SAM Children

Out of seven deaths, five were male (71.4%). Female SAM children in our study population were the majority (58.7%). Mothers responded to the calls the most (44.8%), whereas Sahiyas and ANMs responded to 25% of the calls made. The majority of the SAM children were found to be the first in the family order (45.3%), of which female children comprised 28.3%. Few calls (5.3%) were answered by other family members, including uncles, aunts, or close relatives of the parents (Tables 1, 2).

**Table 1:** Showing the details of all SAM children including present living status, relationship with respondents, birth order and age category N=407 n (%): n-in number, (%)-in percentage, mo-months, yr-years Note: no child older than 2 years or third child in the family among the dead.

Variables	Child Status		Female Male		Total	p-value
Age Category	Alive	<6mo	11 (2.8)	8 (2.0)	19 (4.8)	0.378
		>6mo - <2yr	175 (43.8)	109 (27.3)	284 (71.0)	
		>2yr	52 (13.0)	45 (11.3)	97 (24.3)	
	Dead	<6mo	1 (14.3)	0 (0.0)	1 (14.3)	0.008
		>6mo - <2yr	0 (0.0)	6 (85.7)	6 (85.7)	
Relations	Alive	Mother	107 (26.8)	72 (18.0)	179 (44.8)	0.171
		Father	49 (12.3)	36 (9.0)	85 (21.3)	
		Family	15 (3.8)	6 (1.5)	21 (5.3)	
		Shaiya	26 (6.5)	21 (5.3)	47 (11.8)	
		ANM	34 (8.5)	15 (3.8)	49 (12.3)	
		Others	7 (1.8)	12 (3.0)	19 (4.8)	
	Dead	Father	1 (14.3)	0 (0.0)	1 (14.3)	0.072
		Family	0 (0.0)	3 (42.9)	3 (42.9)	
		Shaiya	0 (0.0)	2 (28.6)	2 (28.6)	
		ANM	0 (0.0)	1 (14.3)	1 (14.3)	
Birth Order	Alive	First child	114 (28.3)	68 (17.0)	182 (45.3)	0 .160
		Second child	59 (14.8)	54 (13.5)	113 (28.3)	
		Third Child	28 (7.0)	25 (6.3)	53 (13.3)	
		Fourth Child	17 (4.3)	8 (2.0)	25 (6.3)	
		Fifth Child	20 (5.0)	7 (1.8)	27 (6.8)	
	Total		238 (59.5)	162 (40.5)	400 (100.0)	
	Dead	First child	1 (14.3)	3 (42.9)	4 (57.1)	0.831
		Second child	0 (0.0)	1 (14.3)	1 (14.3)	
		Fourth Child	0 (0.0)	1 (14.3)	1 (14.3)	
		Fifth Child	0 (0.0)	1 (14.3)	1 (14.3)	
	Total		1 (14.3)	6 (85.7)	7 (100.0)	

**Table 2:** Various aspects of counseling and information provided before SAM children’s discharge N=400 n (%)

Relatives of all Alive SAM Children		Relationship							p-value
		Mother	Father	Family	Shaiya	ANM	Others	Total	
Counseling at the time of discharge	Yes	172(96.60)	77(90.60)	18(85.70)	46(97.90)	45(90.00)	19(100)	377(94.30)	0.057
	No	6(3.40)	8(9.40)	3(14.30)	1(2.30)	5(10)	0	23(5.80)	
Information Provided under various headings	Others	13 (3.3)	11 (2.8)	2 (0.5)	6 (1.5)	5 (1.3)	2 (0.5)	39 (9.8)	0.375
	Hygiene	119 (29.8)	52 (13.0)	12 (3.0)	29 (7.3)	31 (7.8)	15 (3.8)	258 (64.5)	
	All Info.	43 (10.8)	15 (3.5)	5 (1.3)	12 (3.0)	9 (2.3)	2 (0.5)	85 (21.3)	
	No Info.	4 (1.0)	7 (1.8)	2 (0.5)	0 (0.0)	4 (1.0)	0 (0.0)	17 (4.3)	
Awareness regarding breastfeeding if the mother is COVID-19 positive	Yes	50 (12.5)	28 (7.0)	4 (1.0)	24 (6.0)	20 (5.0)	6 (1.5)	132 (33.0)	0.032
	No	129 (72.10)	57 (67.10)	17 (81.0%)	23 (48.90)	29 (59.20)	13 (68.40)	268(67.0)	
Total		179 (44.8)	85 (21.3)	21 (5.3)	47 (11.8)	49 (12.3)	19 (4.8)	400 (100.0)	

Counseling of caregivers

We looked at the different parts of the nutritional counseling services given to parents when their children were leaving MTCs. During the COVID-19 pandemic, we also tried to find out if they were given the right information about breastfeeding and other ways to feed their babies. Other areas assessed include food quantity and diversity, feeding frequency, hand hygiene, child immunization, danger signs, and COVID-19 prevention through maintaining social distance and the use of face masks. The majority of parents (93.8%) were counseled at the time of discharge. Only 21.3% said they were counseled on all aspects of proper feeding practices. Counseling regarding the fact that a COVID-19-positive mother can still breastfeed her baby was not given to the majority (given only to 12.5%), which was statistically significant with a p-value <0.05 (Table 2).

Nutritional and health status of children under the age of six months

All children aged six months and younger are expected to be exclusively breastfed (EBF). Out of 20 such children, one died, and one case did not provide any response among the 19 alive SAM children. Five parents responded that their children were not on EBF, and all were female. Two respondents did not give the SAM children EBF because they did not have sufficient breast milk production. The remaining three girls were also found to be thin. Other responses regarding EBF that we found in striking contrast among boys were that 55.60% were breastfed >eight times a day while 40% of girls were breastfed <eight times a day. Only a few caretakers observed post-discharge ailments (15.4%) and weight loss (7.7%) in their children while tending to exclusively breastfeed the boys most frequently. This was statistically significant with a p-value <0.05 (Table 3). 

**Table 3:** Children alive (N=400) categorized in various age categories and their feeding practices

Age Group	Questions		Male	Female	Total	p-value	
Age 0-6 months (n=19)	Number of times breastfeeding was given a day	No breastfeed	1(11.10)	4(40.00)	5(26.30	0.165	
		>8 Times	5(55.60)	1(10.0)	6(31.6)		
		5-6 times	2(22.20)	4(40.0)	6(31.6)		
		<2 times	1(11.10)	1(10.0)	2(10.50)		
	Post-discharge complications	Yes	1(11.10)	2(20.00)	3(15.80)	0.596	
		No	8(88.90)	8(80.00)	16(84.20)		
	Is the child thin now?	Yes	2(22.20)	2(20.00)	4(21.10)	0.906	
		No	7(77.80)	8(80.00)	15(78.90)		
	Total		9(100)	10(100)	19(100)		
Age >6 months to <2 Years ( N=283)	Number of times breastfed	No Breast Feed	12(11.10)	21(12.00)	33(11.70)	0.45	
		More than 8 times	30(27.80)	49(28.0)	79(27.90)		
		5/6 times	47(43.50)	83(47.40)	130(45.90)		
		2-3 times	15(13.90)	21(12.00)	36(12.70)		
		<2 times	2(1.90)	0	2(0.70)		
		Others	2(1.90)	1(0.60)	3(1.10)		
		Total	108(100)	175(100)	283(100)		
	Post-discharge complications	Yes	21 (19.4)	26 (14.9)	47 (16.60)	0.314	
							No	87 (80.60)	149 (85.10)	236 (83.40)		
		Child health complaints?	Yes	14(13.00)	9(5.10)		23(8.10)	0.019		
	No		94(87.00)	166(94.90)	260(91.90)			
	Eating normally?	Yes	102(94.40)	170(97.10)	272(96.10)	0.254		
		No	6(5.60)	5(2.90)	11(3.90)			
	Number of times eating per day	1	2 (0.7)	1 (0.4)	3 (1.1)	0.254		
		2	11 (4.0)	11 (4.0)	22 (7.9)			
		3	76 (71.70.5)	141(82.90)	217(78.60)			
		4	17 (16.0)	17 (10.0)	34 (12.30)			
	Total food groups	No response	3 (2.78)	6 (3.42)	9 (3.2)	0.707		
		1	8 (7.40)	10 (5.71)	18 (6.3)			
		2	26 (24.07)	42 (24)	68 (24.02)			
		3	31 (28.70)	46 (26.28)	77 (27.20)			
		4	16 (14.81)	28 (16)	44 (15.54)			
		5	10 (9.25)	20 (11.42)	30 (10.60)			
		6	10 (9.25)	21 (12)	31 (10.95)			
		7	4 (3.70)	2 (1.14)	6 (2.12)			
	Total		108 (100)	175 (100)	283 (100.0)			
	Age >2yrs ( n=98)	Post-discharge complications	Yes	3 (3.1)	6 (6.2)	9 (9.3)	0.064		
No			57 (58.8)	31 (32.0)	88 (90.7)			
Is the child thin now?		Yes	9 (9.3)	7 (7.2)	16 (16.5)	0.613		
		No	51 (52.6)	30 (30.9)	81 (83.5)			
Child eating normally?		Yes	56 (58.9)	35 (36.8)	91 (95.8)	0.643		
		No	2 (2.1)	2 (2.1)	4 (4.2)			
Number of times eating per day		2	1 (1.1)	1 (1.1)	2 (2.1)	0.947		
		3	43 (45.7)	28 (29.8)	71 (75.5)			
		4	13 (13.8)	8 (8.5)	21 (22.3)			
Total food groups		No Response	1 (2.20)	3 (5.80)	4 (4.1)	0.256		
		1	1 (2.1)	1 (1.9)	2 (2.1)			
		2	9(19.60)	8(15.40)	17 (17.30)			
		3	8(17.40)	8(15.40)	16 (16.30)			
		4	10 (21.70)	11 (21.20)	21 (21.40)			
		5	7 (15.20)	7 (13.50)	14 (14.30)			
		6	3(6.50)	12 (23.10)	15 (15.30)			
		7	7 (15.20)	2 (3.80)	9 (9.20)			
Total		46 (46.93)	52(53.06)	98 (100.0)			

Nutritional and health status of children aged six to 24 months

We asked respondents about their child's health and nutrition if children were older than six months. They were asked if they thought their child was thinner, sick, eating normally, and feeding frequently, as well as what food groups were served. Girls made up 61.83% of 283 SAM six-month-olds. Overall, 12.0% of girls were not breastfed, whereas 45.90% of SAM kids were breastfed five to six times a day. According to the respondents, 83.40% had no complications after discharge, whereas 16.60% had a fever, diarrhea, lethargy, or other illnesses. Most parents said their child ate well, but only 12.3% ate more than four times a day. Only 39.11% of SAM children received four or more types of food, indicating a lack of food diversity. Most children denied having a health issue in the past 24 hours. Gender was associated with the nutritional and health status of the child with statistical significance as the p-value was <0.05 (Table 3).

Nutritional and health status of children older than 24 Months

Out of 98 SAM children older than two years, 91 (95.8%) of them, according to the respondents were reported to be eating normally but only 21 (22.3%) of them were found to be eating four or more times a day. On further probing regarding food diversity practices, we found only 59 children received food from four or more groups over the last 24 hrs. According to the respondents' account, more children older than two years were growing without post-discharge complications (90.7%) and without physical thinning (83.5%) in addition to eating normally (95.8%) at most three-four times a day (97.8%), as provided with diverse food (60.2%) (Table 3).

 Past treatment history and feeding practices 

Questions were asked about whether the child was previously admitted to MTCs and then evaluated for the frequency of feeds or breastfeeding if admitted. The age group of the child that was the focus of this study, was those children who were younger than two years but older than six months. Overall, 51 SAM children were previously admitted to MTCs, out of which close to three-quarters (73.58%) of them were younger than two years old, and only one-fifth (21.6%) of these children were being fed four times a day (Table 4). It is also worth mentioning that the caregivers alluded to dissatisfaction regarding the distribution of take-home rations (THR) in most instances. A multiple logistic regression model was applied for all living children across the three age groups, to learn what predictors might decide whether they were fed according to the maternal infant and young child feeding practices being taught in MTC at the time of discharge. For age groups, less than six months, breastfeeding more than eight times was considered to be well-fed, while for children of age, more than six months but less than two years, and children more than two years, children having more than six food groups were considered to be well-fed. Three predictors were seen to be of statistical significance and were considered major predictors in our study. Age in months, (OR 0.55, 1.00-1.11), age category (OR 4.33, 1.71-10.84), and the number of breastfeeds (OR 1.85, 1.07-3.21) were the three predictors. While other predictors were not of statistical significance like gender, the relationship of the respondent, birth order, MTC previous visit, counseling at the time of discharge, etc (Table 5).

**Table 4:** Association of Previous MTC visits, Age Category and Feeding patterns N= 400.

Age Category. Feeding practices			MTC Previously visited		Total	p-value
			Yes	No		
>6mo- <2yr ( 7 no response ). N=283	Times eating a day	1	1 (2.54)	2 (0.84)	3 (1.08)	0.409
		2	4 (10.25)	18 (7.59)	22 (7.97)	
		3	27 (69)	190 (80.16)	217(78.62)	
		4	7 (17.94)	27 (2.95)	34 (12.31)	
	Total		39 (100)	237 (100)	276 (100.0)	
>6mo- <2yr N=283	Breastfeeding status	No breastfeed	6(15.38)	27(11.06))	33(27.91)	0.011
		More than 8 times a day	11(28.20)	68(27.86)	79(11.66)	
		5/6 times	14(35.89)	116(47.54)	130(45.93)	
		2-3 Times	6(15.38)	30(12.29)	36(12.72)	
		<2 Times	2(5.12)	0	2(.70)	
		Other sources of milk	0	3(1.22)	3(1.06)	
	Total		39(100)	244(100)	283(100)	
>2yr ( 3 No Response) N=98	Number of times eating a day	2	0 (0.0)	2 (3.2)	2 (3.2)	0.084
		3	9 (9.5)	63 (63.2)	72(74.7)	
		4	3 (3.2)	18 (18.9)	21 (22.1)	
	Total		12 (100)	83 (100)	95 (100.0)	

**Table 5:** Multiple logistic regression analysis for outcome as well fed and other dependent factors as predictors (N=400)

Variables	Adjusted OR	95% C.I	
Gender	0.518	0.184	1.463
Relationship	1.304	0.904	1.881
Birth order	1.396	0.838	2.326
Number of breastfeeds*	1.859	1.074	3.217
MTC previous visit	1.285	0.338	4.887
Counseling at the time of discharge	0.46	0.092	2.305
Child health now	0.845	0.089	7.986
Child thin now	0.256	0.029	2.24
Age in months*	0.55	1.00	1.11
Age category*	4.332	1.715	10.945

## Discussion

Mobile phone outreach to health beneficiaries has been widely advocated but with mixed results [20, 22, 23]. COVID-19 severely impacted most of the world, including India [24, 25]. Data retrieval via phone or mobile is always difficult [26, 27]. Despite efforts to avoid cultural, language, and perspective gaps, phone data will still be flawed. However, during the COVID-19 pandemic, beneficiaries could be reached by phone or mobile.

Our COVID-19-era Jharkhand MTC study found only seven SAM child deaths out of 407 participants, confirming other studies. MTC program in India has evidenced high survival outcomes (<5% child deaths) [9,28]. Additionally, 85% of SAM children-mostly girls (54.6%)-reported no complications after discharge. Previous Indian and Jharkhand studies also found similar post-discharge morbidity. In our study, 16.5% of SAM children had post-discharge health issues like fever, diarrhea, lethargy, or other illnesses, with diarrhea being the most common [9, 29, 30]. Micronutrient deficiencies may cause post-discharge co-morbidities. Our study found many children received less frequent and less diverse food, so micronutrient deficiencies may contribute to these post-discharge morbidities [9].

Nearly 25% of the respondents were Sahiyas or ANMs who referred SAM children to MTCs or worked at those MTCs. Indicating the children's parents either did not have phone numbers or parents did not give their numbers for public use. This discovery is new in similar settings. It may indicate that impoverished rural Jharkhand may lack intent, training, or mobile phones. When asked about the relationship of the respondents to the SAM children, we found that close to 1/4 of respondents were either Sahiyas or ANMs who either referred the SAM children to the MTCs or were themselves staff members of those MTCs. This finding is novel, as it has not been documented in similar settings. This may show the possible reasons behind this finding could be a lack of intent, training, or mobile phones seen in the impoverished rural hinterlands of Jharkhand. Once a SAM child is admitted to the MTC, the mother is also provided with training to maintain hygiene and feed quality or quantity while the child is in treatment [7, 9, 10]. During the mobile interviews, an effort was made to document training details by asking them direct questions like what, how, and on which aspects they were trained. It was found that although counseling was given, it was not done in its entirety as expected. Our study revealed just 21.3% of COVID-19 counseling was complete. Studies done in the pre-COVID-19 era found the poor capacity building of mothers at the MTCs was linked to the frontline health workers themselves, who exhibited a lack of good explanations about faltering growth, had knowledge gaps, and showed limited skills transfer [28, 29]. We found children who were discharged from MTCs were not fed according to the accepted norms and guidelines. Additionally, we found gender does play a role, even in the decision of whether to breastfeed or not. The integrated child development services (ICDS) program ensures that SAM children once discharged and back in their homes should get double the amount of THR, but, due to COVID-19 restrictions, most of the blocks were not providing THR thereby denying the SAM children their extra bite of nutrition [25].

This study has several limitations as it was done solely based on the available mobile phone records on the malnutrition treatment centers-monthly information system (MTC-MIS) website which might not contain an exhaustive database. Caregiver’s responses to questions pertaining to comparative “thinning," feeding frequency, and post-discharge complications are subjective assessments that are prone to recall bias and inaccuracies. This study also lacks information on the lack of quantifying weight change and daily nutritional intake against the requirements of age-specific standards.

## Conclusions

Our study highlights the various gaps in the system of caring for SAM children in Jharkhand. Stronger follow-up care of discharged SAM children through collaboration at the community level is as important as regular contact at the facility level. Community involvement through Sahiyas and ANMs is critical to minimize the relapse of malnutrition. Basic counseling should be delivered on several issues including EBF for the first six months of life, appropriate post-discharge diet, detailed instructions on hygiene, food diversity, and frequency along with watching for danger signs. At the time of discharge, parents should be given counseling materials with pictorial depictions of how to take care of children at home. COVID-19 obviously posed an additional burden on the management of such vulnerable children. Counseling for proper methods for lactation/child-feeding, if the mother or child were COVID-19 positive, was lacking.

Although the malnutrition management system enabled lower post-discharge complications, maintained the regained body weight, and recorded fewer fatalities; there still exists a need to achieve more in terms of food diversity, feeding frequency, and correct practice. System-wide collaborations and further studies addressing household norms and community beliefs will have greater input in improving malnutrition care. We advise that more field studies be conducted to validate our findings which will strengthen the existing system if addressed properly.
